# Evaluating Simulated Consultation Videos in Teaching Patient-Centered General Practice

**DOI:** 10.15694/mep.2020.000033.1

**Published:** 2020-02-17

**Authors:** Merete Jorgensen, Klaus Witt, Marjukka Makela

**Affiliations:** 1 Center for Education and Research in General Practice. Copenhagen University

**Keywords:** medical undergraduate student, patient-centered consultation model, assessment

## Abstract

This article was migrated. The article was not marked as recommended.

**Introduction:** In the General Practice course at Copenhagen University, students are taught the patient-centered consultation. The aim of this study is to evaluate the feasibility of a new method for measuring the effect of this teaching and of adding access to simulated consultation videos to usual teaching.

**Methods:** The University assigned 293 final-semester students to three groups: a Control group with usual curriculum, an Access group that watched simulated consultation video clips online and a Teaching group where the video clips were discussed in teaching sessions. The outcome was change in students’ ability to identify patient-centered elements in a test video, measured with a questionnaire before and after the course.

**Results:** An overall teaching effect was observed, which was most apparent in communication items such as making a contract about the topic for the consultation and summarizing. Changes in clinical items and general issues were small.

**Conclusion:** A tool formeasuring the effect of teaching general practice consultation skills combining a test video and questionnaire is presented. Topics needing to be highlighted in teaching could be identified using the tool.

## Introduction

At the University of Copenhagen, the patient-centered consultation as defined by Levenstein and Brown is taught during the final semester clinical course in general practice ( Levenstein *et al.,* 1986) ( Brown *et al.*, 1986). Their model describes four general tasks for the doctor: exploring health, disease and the illness; understanding the whole person; finding common ground; and enhancing the patient-clinician relationship. The teaching is based on the experiential learning model, which has been found effective ( Mc Leod, 2010). This model demonstrates a continuous learning process with four basic elements: concrete experience, observation and reflection, forming abstract concepts and testing skills in new situations ( Kolb and Kolb, 2012).Students at the course are between 25 and 30 years old and thus adults. According to theories about adult learning, the students should affect the content and process of their learning. The teaching should focus on adding to their pre-existing knowledge and skills. It must be practical and centered on problem solving instead of memorizing content ( Knowles, 1980). The course in general practice is based on these two learning principles.

The effectiveness of teaching patient-centered consultation skills to medical students can be evaluated in several ways. Teachers can rate student performance from audio and video recordings ( Burt *et al.*, 2014) ( Cömert *et al.,* 2016). The ratings are dependent on the observer’s goals, preferences, and the quality of the rating scale. Secondly, self-efficacy questionnaires have been used ( Zachariae *et al.,* 2015), but physicians and students are poor assessors of their own performance, as low performers tend to overestimate their own skills ( Davis *et al.,* 2006).A third method is interviewing students individually or in focus groups about the teaching ( Braverman *et al*., 2016). The result is dependent on the interviewer, when and where the interview takes place, and the right items ( Berk, 2005).

Teachers and curriculum planners must react to new types of students arriving, the “Digital Natives” who have grown up with mobile phones, tablets and constant access to internet ( Prensky, 2001). Flipped classroom (FC) moves homework into the classroom as the students prepare for teaching sessions at home ( Abeysekera and Dawson, 2015). Current evidence suggests that the use of flipped classroom in health professions education makes a significant improvement in student learning compared with traditional teaching methods ( Hew and Lo, 2018).

The aims of this study were to evaluate the feasibility of a new method for evaluating the effect of teaching patient-centered consultations and evaluate the effect of adding simulated consultation videos to usual teaching in a FC design.

## Method

An open prospective cohort study was conducted using a simulated consultation video and a questionnaire before and after the course of General Practice at the University of Copenhagen (KU) in 2013.

The teaching

Teaching General Practice at KU is taught in the first and final semesters. Kolb’s experimental model and Knowles’ four adult learning theories are used in planning the curriculum at the final semester (see Table 1).

**Table 1.** Teaching in General Practice. Knowles’ four adult learning strategies applied to the course in General Practice at Copenhagen University

Teaching in General Practice		
Theory	What it means for the student	What it means for the teacher
Self-directed learning	The student knows what he/she needs to know	The teacher needs constant evaluation to adapt to learning needs
Experiential learning	The student performs in a GP role and gets feedback. Integrating existing knowledge into a new role	Alternating work in a GP clinic and feedback sessions gives the teacher the possibility to address the student’s actual needs
Relation to a work situation	Learning is related to a specific context (GP clinic)	Feedback to students on their own consultation videos, discussing patient-centered elements
Problem-based learning	The student deals with complex symptom presentation from patients in General Practice	In the small group sessions clinical and communicative problems are discussed with the teacher and peers

GP: general practitioner

Students were invited by e-mail before the course to watch a ten-minute lecture about patient-centered consultations on the learning platform. The students were familiar with the platform, as all communication between the university, the teachers and the students is handled via this platform. On the first day of the course, the authors gave a one-hour lecture on communication.

Final-semester students work eight days in a General Practice clinic, where they have consultations with real patients, video-record these consultations and receive feedback from their tutor general practitioner (GP). In small group sessions at the university, they discuss their videos with their peers and a teacher (twenty hours in all). The small group sessions and lectures alternate with days in General Practice. The teachers were 17 trained GPs with a special interest in teaching, eight women and nine men. The university employed six and eleven were associated teachers. The teachers were the same during the study period. For the oral exam, students select one of their own consultation videos to present for analysis. The students are assessed by their performance in the video and their ability to analyse it according to the patient-centered method. They are presented with a clinical case for discussion and a theoretical question about the role of General Practice in the primary healthcare sector.

### The questionnaire

The DanSCORE questionnaire (Danish Structured Consultation Observation Registration and Evaluation) was originally developed for research purposes. It was designed by one of the authors (KW) to evaluate a whole consultation in General Practice.

The questionnaire has 33 items. Fifteen items reflect communication skills (items 1-7, 10, 14-20), five items clinical skills (8-9, 11-13) and thirteen (21-33) are about the consultation in general (see Supplementary Material 1). The general items dealt with topics related to the structure and duration of the consultation, doctor’s role, use of understandable language, interrupting the patient, and reaction to patient mood; these are observable and not directly related to the original patient-centered method.

The questionnaire was piloted in two studies in 2012. In the first pilot study, 13 final-semester students used the questionnaire to analyse their own videos in a teaching session. They assessed each element and its response categories for comprehensibility. Minor linguistic corrections were made. In the second pilot study, 45 final-semester students watched the test video before and after the General Practice course, completed the questionnaire and commented on the questionnaire as a whole. Again, minor text corrections were made. For analyses, responses were collapsed into two categories (correct or incorrect).

A simple framework to help medical students master the patient-centered elements working in General Practice was developed (see Table 2).

**Table 2.** Framework for students working in General Practice: structure and elements to observe in a consultation

Structure for medical student consultations in General Practice
**In the patient’s part**
an agreement about the agenda of the consultation
the patient’s ideas about the symptoms
the impact of illness on daily functions
feelings aroused by the symptoms
the patient′s expectations regarding the consultation
**In the doctor’s part**
history taking based on hypotheses formed during the patient’s narrative
clinical problems appropriately considered
**In the joint part**
mutual agreement about diagnosis is reached
mutual agreement about plan is reached
doctor informs the patient about the safety net
**In the general part**
appropriate use of time in the consultation
the doctor informs the patient about what happens next in the consultation (signposting)
the doctor’s attentiveness to the patient
summarizing to obtain shared understanding
consultation structure

#### Test video

The test video of 15 minutes shows a consultation between a general practitioner (GP) and a simulated patient. The GP (university teacher) was informed that the consultation would be video-recorded for teaching purposes but knew nothing about the patient and the symptoms. The simulated patient played a stressed person with a headache and was instructed to act like a real patient.

The teachers, all experienced GPs, had difficulty in evaluating the video from the perspective of a medical student, and their replies differed to some extent. To reach consensus on correct answers for the test video, a modified Delphi method was used. There was still disagreement regarding five items, so the authors decided to transcribe the test video. Two authors (MJ, KW) then set the criterion standard, making a final decision on correct replies. For two items, two replies were accepted as equally correct because the response categories overlapped.

### Simulated consultation clips

For teaching purposes, 16 brief video clips (0.35-5.36 minutes) with three different simulated patients and two authors (MJ, KW) in the GP role were produced. The teaching material could be accessed anywhere by mobile phone or tablet, so patient anonymity could not be ensured. Therefore, SPs (Simulated Patients) were used.

To keep consultations as authentic as possible, scripts were not used, and actors were instructed to present symptoms they were familiar with. The doctors were comfortable in their roles and varied their behavior to increase learning potential. A professional company used two cameras and the “first takes” of all 16 simulated consultations were successful. The clips were edited on the spot, based on whether the doctor’s or the patient’s face was to be shown. Apart from that, no editing was done. Each video was followed by questions about elements of the patient-centered consultation. The students received no feedback, as the simulated consultation clips were meant to lead to reflection. The students’ use of simulated consultation clips was measured electronically on the learning platform.

### Interventions

Final-semester students in 2013 were placed by the University in three teaching groups. The first of these was a “Control” group, receiving the usual teaching; the next group (“Access”) also had access to the simulated consultation clips. The students in the third (“Teaching”) group were asked by the teachers to watch four simulated consultation clips before each small group session for discussion. The teachers’ main priority was still to give feedback on students’ own consultation videos recorded during their work in General Practice. After the course, teachers were asked how many video clips they had discussed in their small groups.

### Testing

On the first day of the course, all students watched the test video during a lecture and completed the questionnaire. Five weeks later, on the last day of the course, the procedure was repeated. Students were not allowed to talk while completing their questionnaire. Each student’s pre-course and post-course questionnaires were linked, without revealing students’ identities.

### Statistics

The students’ answers were evaluated as either correct or incorrect. They were placed in four groups according to changes between the first and last session: Incorrect-Incorrect, IncorrectCorrect, Correct-Incorrect and Correct-Correct. A teaching effect was calculated for each item as the percentage of students that improved, e.g. changed from incorrect answer before the course to correct answer after the course. Group differences between the use of video clips and between examination marks were calculated by an X2-test.

## Results

All 293 students in the three groups were included. Pre-course and post-course data were available for 217 students (74 %) who completed both questionnaires ( Table 3).

**Table 3.** Participants, number and gender

Study participants			
Groups	Control group	Access group	Teaching group
Participants in the course	85	106	102
Female	56 (66%)	74 (70%)	68 (67%)
Replies from both pre- and post-test	69 (81%)	77 (71%)	71 (70%)

Students who did not participate in the post-course test had similar answers in the pre-course test as those who completed both tests.

The teaching” group watched more clips on average than the “Access” group, but the difference was not significant (p = 0.07). The video clips were to be watched before four teaching sessions (out of five). The teachers reported having discussed eight of the 16 simulated consultation clips on average in the small group sessions (see Table 4).

**Table 4.** Use of video clips. The topics and duration of the simulated consultation clips

Students’ use of video clips			
Topics in the video clips	Duration of video clips	Number of students who accessed the video	
Groups		Access (n=106)	Teaching (n=102)
**Used before teaching session 2**		n (%)	n (%)
Patient with stress 1	4′21″	56 (53)	76 (75)
Young mother with pain in joints	4′19″	14 (13)	60 (59)
Headache 1	4′30″	1 (1)	47 (46)
A good solution?	1′45″	23 (22)	40 (39)
**Used before teaching session 3**			
Doctor and patient disagree	0′40″	24 (23)	31 (30)
Young woman with a knee problem	2′47″	18 (17)	30 (29)
Headache 2	4′29″	23 (22)	26 (25)
When an important thing is missing	1′30″	32 (30)	29 (28)
**Used before teaching session 4**			
Patient with stress 2	3′33″	29 (27)	19 (19)
What about a sick leave?	1′27″	21 (20)	15 (15)
A dizzy and demanding patient	5′36″	1 (1)	18 (18)
An unacceptable solution	1′09″	19 (18)	17 (17)
**Used before teaching session 5**			
Patient with stress 3	1′04″	24 (23)	17 (17)
When something has been forgotten	0′35″	18 (17)	17 (17)
Young woman with arthralgia	3′14″	11 (10)	29 (28)
A dizzy, demanding and worried patient	4′08″	26 (25)	6 (6)
**Total number of video clips used**		**338**	**477**
**Average use per student**		**3,2**	**4,7**

The teaching effect was clearest in communication items, but mostly lower in clinical and general items (see Supplementary Material 2).The change in students’ ability to identify communicative consultation skills (patient-centered elements) are seen in Table 5.

**Table 5.** Change in students` ability to identify communicative consultation skills

Teaching effect in communication items			
Groups	Control	Access	Teaching
Questions	(%)	(%)	(%)
1. Agreement about the *topic for the consultation*?	48	36	41
2. Patient`s *ideas* explored?	25	14	13
3. Patient`s *function* explored?	10	22	18
4. Affect the patient`s *self-image*?	15	29	13
5. Are the patient′s *feelings* explored?	19	24	28
6. Are the patient`s *expectations* explored?	19	18	18
7. Does the doctor *summarize* the patient’s history?	27	31	31
10. Does the doctor *summarize* the diagnostic interview?	34	29	40
14. Does the patient makes an *informed decision*?	27	19	12
15. The patient understand the doctor`s *conclusion*?	24	24	19
16. The patient understands the *indication* for further tests?	19	18	35
17. The patient understands the *plan* for treatment?	40	13	0
18. The patient summarizes the *diagnosis and plan?*	16	8	17
19. The patient know what to observe and how to react? *(Safety-net)*	12	23	17
20. Is the patient asked to *summarize theinstructions?*	1	0	10

The teaching effect is calculated as the percentage of students changing from an incorrect answer before the course to a correct answer after the course. In almost all questions, a positive teaching effect is seen. The teaching effect can be small if teaching is insufficient or if students know about the topic beforehand, so there is little room for change.

In all three groups, a clear teaching effect was observed, especially in three items: “making a contract about the topic for the consultation” (item 1) and “summarizing” (items 7 and 10). In five items, the change in ability to identify communicative consultation skills was low:

• asking about patient expectations (item 6)
• informing the patient (items 15 and 16)
• safety netting (item 19)
• asking the patient to summarize the instructions (item 20)

The students had only minor problems evaluating the clinical items, with small variations between the groups. They agreed that the doctor’s conclusion was likely (item 11) and that the decision about treatment was medically correct (item 13) (see Supplementary Material 2). The change in ability to identify general issues was relatively low and varied between the groups (see Supplementary Material 2).No significant differences in examination grades between the groups were observed (see Table 6).

**Table 6.** Exam grades

Performance description	Grades	Control	Access	Teaching	
	Danish	English	N=86	N=106	N=101
Performance that is unacceptable in all respects.	-03	F	0 %	0 %	0 %
Performance which does not meet the minimum for acceptance	0	F ^x^	1 %	0 %	5 %
Performance meeting only the minimum requirements for acceptance	2	E	0 %	4 %	1 %
Fair performance displaying some command of relevant material but also some major weaknesses	4	D	3 %	4 %	4 %
Good performance displaying good command of the relevant material but also some weaknesses-	7	C	21 %	24 %	21 %
Very good performance displaying a high level of command of most aspects with only minor weaknesses	10	B	41 %	33 %	38 %
Excellent performance displaying a high level of command of all aspects with none or few weaknesses	12	A	34 %	36 %	32 %

No significant difference in grade distribution is seen (X2 > 0.05). One student in the teaching group did not participate in exam due to illness.

## Discussion

### Strengths and weaknesses

No convincing effect of introducing simulated consultation videos in the teaching was seen, but students’ ability to identify communication items was improved. The study was controlled but not randomized or blinded. A randomized controlled study would have been ideal, but this design is rare in educational studies ( Norman G and Eva K, 2014). In our study, it would have been impossible to blind the groups, as students share teaching materials across groups. The study was done in a real teaching environment while developing teaching methods. Other strengths include stepwise introduction of the online material, online registration of video use, stability in the teachers’ group, and good follow-up of participants.The test video as such could not produce intensified learning since a consultation video has for years been shown the very first day of the course in General Practice at the University of Copenhagen ( Spreckelsen and Juenger, 2017). If existing rating scales or observation guides had been used instead of the questionnaire, it might have overwhelmed undergraduate medical students. For example, The Cambridge-Calgary observation guide has more than 70 items ( Kurtz and Silverman, 1996) ( Burt *et al*., 2016).

All three groups gave mostly similar pre-course answers, indicating that students found the items and response categories understandable. For the test video, two items could be answered in several correct ways. Regarding the contract about the agenda (item 1), the doctor says, “So you come with a headache?” and the patient nods. We teach students to ask if the patient has other topics to be addressed, so “no” would be our preferred answer, but the nod makes “partly” in this case also acceptable.

For the item on the doctor interrupting the patient (item 28), the response categories were “no”, “yes but acceptably”, and “yes, unacceptably”. The doctor interrupts with half a word, so a “no” and a “yes but acceptably” are equally correct.

In the small group sessions, students watch a number of each other’s videos, so they have probably forgotten the test video when they see it again. Results might have been different if we had used another test video at the end, but it could have led to a discussion of which video was easiest to observe.

The students entering the course already had experience working with patients so taking history and examining patients were well known to them. Many had problems evaluating the information given to the patient in the test video (item 15, 16, and 17). This could be expected, as primary care is a new environment. The epidemiology and handling of diseases are quite different from hospital settings ( Braverman *et al.*, 2016) ( Boggiano *et al.,* 2017).

The teaching effect was best in communication items, as the patient-centered consultation was new to students. They were not accustomed to ask for patient expectations, as their medical experiences came from hospitals, where the patients not usually are asked about this. The same is the case for safety netting and for letting the patient summarize the instructions.Very few students went from correct to incorrect answers during the study. The duration of the short simulated consultation clips of up to five minutes seemed appropriate ( Brame, 2016). The overall use of clips, however, was much lower than expected. The teachers were asked, after the course, how many simulated consultation clips they had discussed with their students. A recall bias may exist, they may not wish to reveal their technical deficiency, or they may simply have tried to please the investigator. On the other hand, introducing a new teaching method requires some time before optimal use is found ( Thorell *et al.*, 2015). The students were also somewhat unfamiliar with the flipped classroom concept.

As opposed to students as “Digital Natives”. teachers are “Digital Immigrants” and teach in an old-fashioned way ( Prensky, 2001). This could explain the teachers’ reluctance to embrace online material. The teachers also experienced lack of time for important structured personal feedback and only used half of the planned videos.

The term “digital natives” probably comprises a heterogeneous group of students, variations due to upbringing, nationality, and access to technological equipment (Watson, 2013). Our study population is a homogeneous group, as Denmark is one of the most digitalized countries in Europe (Eurostat, 2015 https://ec.europa.eu/eurostat).

Students’ motivation is related to their need for new skills, and they benefit from applying new knowledge immediately. Alternating clinical work and feedback in small group sessions contributes to this, and the need comes from taking on a new role as GP ( Peters *et al.*, 2017). Evaluating students’ ability to analyse consultations is a simplified proxy of learning the complexity in General Practice, but learning is a stepwise process and analyzing and evaluating are part of the steps ( Adams, 2015).

## Conclusion

The students completing the course in General Practice at KU have learnt to identify important patient-centered elements in a consultation, but the new teaching method was somewhat difficult to implement for teachers as well as for the students.

A new and feasible way to evaluate the effect of teaching General Practice consultation skills, combining a test video and questionnaire, has been presented. Topics needing to be highlighted in teaching can be identified in this way.

## Take Home Messages

• Alternating practice and reflection functions well in teaching general practice
• Implementing online materials for teaching takes time
• Identifying good communication is partly in the eye of the beholder
• The questionnaire was a feasible instrument for measuring teaching effect

## Notes On Contributors

Merete Jorgensen MD.GP. Teaching General Practice to undergraduate medical students and postgraduate. Course instructor in the General Practice Course from 2001-2016. Teaching evidence based medicine in the specialist training.

Klaus Witt MD.GP. Ph.d. Teaching General Practice to undergraduate medical students and postgraduate. Course instructor in the General Practice Course from 1992-2001. Teaching evidence based medicine in the specialist training.

Marjukka Makela GP. Ph.d. M.Sc.National Institute for Health and Welfare, Finland, Helsinki. THL with expertise in Health Economics, General Practice, Medical Technology. Professor in General Practice. Copenhagen University.
