# Supplementary material for: Evaluating Simulated Consultation Videos in Teaching Patient-Centered General Practice
Source: MedEdPublish (2016). 2021 Feb 26;9:33. Originally published 2020 Feb 17. [Version 2] doi: 10.15694/mep.2020.000033.2 (PMC10697464; doi:10.15694/mep.2020.000033.2)
Supplement: Peer review history

# Reviewer report: Reviewer response for version 2

**DOI:** 10.21956/mep.20259.r31437

**Published:** 2021-07-19

**Authors:** Megan Anakin

**Affiliations:** 1 University of Otago

This review has been migrated. The reviewer awarded 4 stars out of 5 Although I did not review the first version of this paper, I have read it and the reviewers comments. I agree with Ken Masters' assessment of this revised article, the authors have addressed the major concerns of the reviewers and produced an article reporting their study of teaching general practice consultation skills that will be of interest to educators elsewhere.

Reviewer Expertise:

NA

No decision status is available

# Reviewer report: Reviewer response for version 2

**DOI:** 10.21956/mep.20259.r31438

**Published:** 2021-03-12

**Authors:** Ken Masters

**Affiliations:** 1 Sultan Qaboos University

This review has been migrated. The reviewer awarded 4 stars out of 5 This is a revised version of the paper. The authors have addressed my major concerns in the previous version. Thank you.

Reviewer Expertise:

NA

No decision status is available

# Reviewer report: Reviewer response for version 1

**DOI:** 10.21956/mep.18997.r27246

**Published:** 2020-05-19

**Authors:** Ken Masters

**Affiliations:** 1 Sultan Qaboos University

This review has been migrated. The reviewer awarded 3 stars out of 5 An interesting paper on evaluating simulated consultation videos in teaching patient-centered general practice. Overall, the work has been well conducted, but there are issues with the paper that need correction:• The authors should be careful about unqualifyingly using the term “Digital Native”, as there is considerable debate around it. I would recommend that they either do not use it, OR acknowledge that it is a debated term. (In the Discussion, this, and “Digital Immigrants” needs to be addressed also.) And saying that you think this is the reason behind the teachers’ reluctance to embrace the online tool puts you in a quagmire – unless you have serious data to support that statement, you are opening yourselves for a flurry of criticism. • The authors should make a little clearer the reasoning behind introducing the simulated videos. i.e. what was the perceived need? Was it simply an experiment to see if it could be done? (At the moment, the only perceived need is the reference to the Digital Native.) I am not saying that there was no need: I am saying that the authors should make the underlying need more obvious. • On this issue I think the problem is that the authors appear to be doing two things simultaneously: testing the feasibility of a new way of teaching, and also testing a new evaluation tool. This can be done, but care must be taken to clearly identify the two aims, keep them separate, and then report on them separately.• When presenting gender demographics it is generally not considered appropriate to present one gender only. All gender options offered to the students should be presented (unless only one gender option was offered to the students, in which case, it would be prudent to explain why).• The Discussion is rather jumbled. It begins with a heading of “Strengths and weaknesses”, but it is difficult to see where this sub-section ends. It appears that (at least) the first 2 paragraphs deal with Limitations. So, the Discussion should rather be restructured into a more conventional format, something along the lines of:o Summary of main activitieso Discussion of Results and relating them back to the current literature on the topico Limitations.Minor:• In the second paragraph after Table 3, there is a quotation mark missing around the word “teaching”.So, I think the experimental work is valuable, but the presentation of the paper needs correction. I look forward to Version 2 of the paper in which these issues are addressed.

Reviewer Expertise:

NA

No decision status is available

# Reviewer report: Reviewer response for version 1

**DOI:** 10.21956/mep.18997.r27245

**Published:** 2020-04-30

**Authors:** Dujeepa D. Samarasekera

**Affiliations:** 1 National University of Singapore

This review has been migrated. The reviewer awarded 3 stars out of 5 Useful study. The authors comment "No convincing effect of introducing simulated consultation videos in the teaching was seen, but students’ ability to identify communication items was improved" is interesting because the impact is through a questionnaire which had either correct or incorrect. I am not generally a fan of bi-modal evaluations as medicine has a lot more "grey" parts or range of performances. However, this study is very relevant in the present times with the COVID crisis. Such training is now encouraged. I would have preferred if the study included a more diverse evaluation scheme and a qualitative component to evaluate the effectiveness. Furthermore, if there is a follow through with SPs or actual patients it would have been more useful for many educators who are now struggling to plan clinical training.

Reviewer Expertise:

NA

No decision status is available

# Reviewer report: Reviewer response for version 1

**DOI:** 10.21956/mep.18997.r27247

**Published:** 2020-02-17

**Authors:** Felix Silwimba

**Affiliations:** 1 University of Lusaka

This review has been migrated. The reviewer awarded 5 stars out of 5 A clearly explained study and appropriate to clinical medical education. with minor adaptations it can be applied to low income settings.

Reviewer Expertise:

NA

No decision status is available
